# Maximal Number of Repetitions at Percentages of the One Repetition Maximum: A Meta-Regression and Moderator Analysis of Sex, Age, Training Status, and Exercise

**DOI:** 10.1007/s40279-023-01937-7

**Published:** 2023-10-04

**Authors:** James L. Nuzzo, Matheus D. Pinto, Kazunori Nosaka, James Steele

**Affiliations:** 1 Centre for Human Performance, School of Medical and Health Sciences, Edith Cowan University, 270 Joondalup Drive, Joondalup, WA 6027 Australia; 2 School of Sport, Health, and Social Sciences, Solent University, Southampton, UK

## Abstract

The maximal number of repetitions that can be completed at various percentages of the one repetition maximum (1RM) [REPS ~ %1RM relationship] is foundational knowledge in resistance exercise programming. The current REPS ~ %1RM relationship is based on few studies and has not incorporated uncertainty into estimations or accounted for between-individuals variation. Therefore, we conducted a meta-regression to estimate the mean and between-individuals standard deviation of the number of repetitions that can be completed at various percentages of 1RM. We also explored if the REPS ~ %1RM relationship is moderated by sex, age, training status, and/or exercise. A total of 952 repetitions-to-failure tests, completed by 7289 individuals in 452 groups from 269 studies, were identified. Study groups were predominantly male (66%), healthy (97%), < 59 years of age (92%), and resistance trained (60%). The bench press (42%) and leg press (14%) were the most commonly studied exercises. The REPS ~ %1RM relationship for mean repetitions and standard deviation of repetitions were best described using natural cubic splines and a linear model, respectively, with mean and standard deviation for repetitions decreasing with increasing %1RM. More repetitions were evident in the leg press than bench press across the loading spectrum, thus separate REPS ~ %1RM tables were developed for these two exercises. Analysis of moderators suggested little influences of sex, age, or training status on the REPS ~ %1RM relationship, thus the general main model REPS ~ %1RM table can be applied to all individuals and to all exercises other than the bench press and leg press. More data are needed to develop REPS ~ %1RM tables for other exercises.

## Key Points

**Table:** 

We applied meta-regression to data from approximately 7000 individuals to update the table of the maximal number of repetitions completed at various percentages of one repetition maximum (REPS ~ %1RM relationship).
Sex, age, and training status did not clearly moderate the REPS ~ %1RM relationship; thus, estimates of mean repetitions and between-individuals variation in the main model table can be applied to most individuals and most exercises.
Numbers of repetitions completed across the loading spectrum were higher in the leg press than bench press; thus, separate REPS ~ %1RM tables were created for these two exercises.

## Introduction

The number of repetitions that individuals can be expected to perform to volitional failure at various percentages of the one repetition maximum (1RM) [i.e., the REPS ~ %1RM relationship] is foundational knowledge in resistance exercise programming. Investigations related to this topic were first conducted in the 1950s and 1960s [1–4] and were eventually followed by two influential studies by Hoeger et al. in 1987 [5] and 1990 [6].

For many years, a table of the REPS ~ %1RM relationship has been published in a commonly assigned strength training textbook (Table 1) [7]. This table has been presented as a general guideline based on a small number of studies [e.g., 5, 6]. To the best of our knowledge, no attempt has been made to reaffirm the table, update it, or consider whether it should be made exercise or population specific. The current REPS ~ %1RM table provides only point estimates for the number of repetitions that individuals might be expected to complete at a given relative load. The table does not incorporate the uncertainty of such estimates, nor does it indicate the expected variation between individuals.

**Table 1:** From a commonly assigned strength training textbook [7], the maximal number of repetitions that individuals have historically been thought to complete at various percentages of the one repetition maximum (1RM) [REPS ~ %1RM relationship]

%1RM	Maximal number of repetitions that can be completed
100	1
95	2
93	3
90	4
87	5
85	6
83	7
80	8
77	9
75	10
70	11
67	12
65	15

Muscle endurance or “strength endurance,” the attribute evaluated by a repetitions-to-failure test at a submaximal loads, may be impacted by sex [8–11], age [12–14], or muscle group [15]. Thus, potential moderating influences of sex, age, and muscle group should be considered when examining the REPS ~ %1RM relationship. Moreover, the current REPS ~ %1RM table is specific to the *concentric* 1RM and *concentric* repetitions-to-failure tests at submaximal loads. This has occurred because resistance exercise equipment such as free weights and weight stack machines involves lifting the same load in the concentric and eccentric phases, and concentric phase strength is ~ 40% less than eccentric phase strength [16]. Some evidence suggests that more eccentric-only than concentric-only repetitions can be completed at equal *relative* loads [17]. Thus, when coupled with the rise in popularity of eccentric resistance exercise and the emergence of eccentric exercise technologies that permit eccentric-only repetitions [11, 18, 19], the possibility that the REPS ~ %1RM relationships might differ between concentric and eccentric muscle actions should be considered. Examination of the above issues seems possible using meta-analytic methods given that numerous papers over the past several decades have included data on repetitions-to-failure tests at various percentages of the 1RM.

Therefore, the purpose of the current study was to perform a meta-regression to estimate the maximal number of repetitions that can be performed at various percentages of the 1RM and the variance between individuals in repetitions completed. More specifically, we aimed to provide an updated and more comprehensive table of the REPS ~ %1RM relationship by incorporating uncertainty of estimates from all available data. A secondary aim was to explore if the REPS ~ %1RM relationship is moderated by exercise, sex, age, training status, and muscle action type. Such information might have implications for resistance exercise prescriptions. For example, it might provide practitioners with a more accurate expectation of how many repetitions individuals can be expected to complete at given relative loads. Exploration of moderators might reveal factors that impact the REPS ~ %1RM relationship, as measured by repetitions-to-failure tests.

## Methods

### Literature Search

**Fig. 1:**
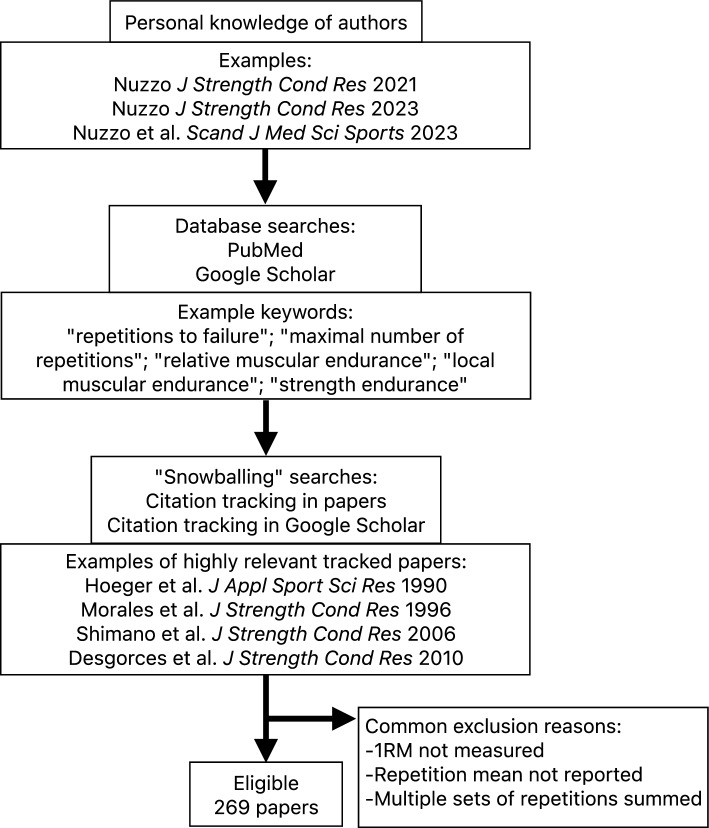
Flow diagram of search strategy. *RM* repetition maximum

Our literature search was thorough, but not necessarily systematic or exhaustive. We used a mixed approach similar to that described by Greenhalgh and Peacock [20] and implementeded in our previous work [16]. The approach relied on the investigators’ personal knowledge [21, 22], checking of personal digital files, relevant keyword searches in PubMed and Google Scholar, and “snowballing” strategies (i.e., reference and citation tracking). Example keyword searches included: “repetitions to failure,” “repetitions to fatigue,” “repetitions to exhaustion,” “number of repetitions,” “maximal number of repetitions,” “muscular endurance,” “strength endurance,” “relative muscle endurance,” “local muscular endurance,” and “task failure.” Searches were performed in January and February of 2023 but were otherwise not limited by publication date. A flow diagram of the search strategy is presented in Fig. 1.

### Eligibility and Data Extraction

A study was eligible for inclusion into the meta-analysis if the following conditions were met: (a) published in English; (b) published in a paper in a journal; (c) human data; (d) the 1RM was tested rather than estimated; (e) a repetitions-to-failure test was performed (i.e., maximal number of repetitions at % 1RM); (f) the test was performed in a non-fatigued state and without concurrent experimental intervention (e.g., blood flow restriction, acute caffeine supplementation, static stretching); and (g) repetitions were reported as unadjusted group means with an accompanying estimate of variance. Both cross-sectional and exercise training studies were eligible for inclusion. With exercise training studies, the extracted data were from baseline/pre-intervention tests. With acute intervention studies, the extracted data were from either pre-intervention tests or from placebo conditions, depending on the study’s design. In studies in which participants performed multiple repetitions-to-failure sets at a given relative load, only data from the first set were extracted, subsequent sets would have been impacted by muscle fatigue. Of note, the data reported by Hoeger et al. in 1987 [5] were later reported in a more extensive paper in 1990 [6]. Thus, only the paper from 1990 was included in the final list of relevant studies.

 Extracted data included sample size, number of study groups tested, study type (e.g., training study), sex, age, body mass, resistance training status and years, exercise, equipment type, 1RM, relative load tested (% 1RM), test pace method (e.g., metronome, self-paced, maximal velocity), repetition duration for the eccentric and concentric phases, and the number of repetitions completed. For age, body mass, resistance training years, 1RM, and number of repetitions completed, the means and standard deviations (SDs) were extracted. The minimums and maximums were also extracted for the number of repetitions completed. Variances reported as standard errors were converted to SDs. For papers in which data were presented in figures, the data were extracted using a graph digitzer (WebPlotDigitizer, https://automeris.io). Finally, some researchers did not report age or body mass for all study groups, but instead reported such data for the entire study sample. In such instances, if the various study groups were all from the same general demographic (i.e., sex and age group), then the values representing the entire study sample were used to represent each study group.

### Statistical Analyses

All extracted data and the analysis code utilized to analyze the data are available at the Open Science Framework (https://osf.io/s94gf/). Given the aim of this research was descriptive, we opted to take a model-based [23] and estimation-based approach [24]. For all analyses, effect estimates and their precision, along with conclusions based upon them, were interpreted continuously and probabilistically, considering data quality, and all within the context of each outcome [25]. Effect size calculation and main modeling was performed using the ‘metafor’ package [26], ‘emmeans’ [27] used for moderator contrasts, and ‘performance’ [28] and ‘bayestestR’ [29] used for model comparison. All analyses were performed in R (version 4.2.2; R Core Team, https://www.r-project.org/) and RStudio (version 2023.03.0 + 492, Posit Software, https://posit.co/). All data visualizations were made using ‘ggplot2’ [30] and ‘patchwork’ [31]. Tables were produced using ‘gt’ [32], ‘gtsummary’ [33], and ‘kableExtra’ [34].

We were interested in modeling the functional form of the relationship between the relative load (i.e., %1RM, predictor variable) and the mean number of repetitions performed and the between-individuals SD in repetitions performed (response variables). As the included studies often had multiple groups and reported multiple repetitions to failure tests at different relative loads within these, the data had a nested structure. Therefore, multilevel mixed-effects meta-analyses were performed with random intercepts for study level, group level, and effect level included in all models. In each model, we allowed for random linear slopes within study and group levels. Effects were weighted by inverse sampling variance. Our initial approach was to examine a selection of different models and compare their fit and performance.

We began with comparing models for both the raw mean repetitions as well as the log-transformed mean repetitions with the predictor taking linear, log-transformed, or quadratic functional forms, and also each model was compared with either the intercept being estimated or with the predictor recentered to force the intercept to take on a value of 1RM at 100% of the 1RM (see visual comparison of these models here: https://osf.io/83c62). It was immediately obvious that the raw means would not be suitable as they permitted the models to predict impossible values (i.e., repetitions < 0). However, the mean repetitions followed a log-normal distribution (see https://osf.io/p8ryh), so we opted to only consider the models of log mean repetitions as candidates. From visual comparison of the log mean models, the linear model appeared to fit the data well. However, the estimated response values at large predictor values of %1RM appeared larger than expected (e.g., ~ 5 repetitions at 95% 1RM). Yet, the recentered models that forced the estimates to take on a value of one repetition at 100% 1RM did not appear to fit the rest of the data well. As such, we examined a final model employing natural cubic splines with knots at 60% and 80% of 1RM (where most data were available; see https://osf.io/qa5gb) and boundary knots at 0% and 100% of 1RM, hoping this model would allow for a good fit to the data available and flexibility to estimate reasonable values at higher values of %1RM. We then compared fit statistics for all log mean models (see https://osf.io/4v32n) and also compared the models using Bayes factors calculated with approximate Bayesian information criterion (see https://osf.io/432gn [35]). Fit statistics favored the natural cubic spline model and Bayes factors indicated that there was strong evidence favoring the natural cubic spline model as being a more probable description of the data generating process compared with all other models. Thus, for log mean repetitions we opted to take the natural cubic spline model forward (diagnostics for this model can be seen here: https://osf.io/e6rqf).

We followed a similar process for comparing models for the variances between people in repetitions performed. In all models, we used the log-transformed SDs for repetitions again with the predictor taking linear, log-transformed, quadratic, or the natural cubic spline functional forms as initially examined for the mean repetitions (see visual comparison of these models here: https://osf.io/wgmrj). Visually, the differences between these models were negligible, which was also confirmed when we compared fit statistics (see https://osf.io/q9brs). Examining the Bayes factors for model comparisons suggested that both the linear and natural cubic spline models had higher probabilities than log-transformed or quadratic; but evidence favoring the natural cubic spline model over the simpler linear model was only marginally positive (see https://osf.io/d87th). As such, for the log SD of repetitions, we opted to take the simpler linear model forward (diagnostics for this model can be seen here: https://osf.io/9kmzg).

A main model including all effects for both log mean repetitions and log SD of repetitions was produced for all groups in each study. From this, we exponentiated the model estimates back to the raw repetition scale to aid interpretability and present meta-analytic scatterplots showing the relationship of both mean repetitions and the SD of repetitions with %1RM with both 95% confidence intervals (CIs) and 95% prediction intervals. We also tabulated the estimated values and CIs for levels of %1RM that range from 15 to 95% (i.e., the range of the data).

As a secondary aim, we conducted exploratory interaction models for both log mean and log SD of repetitions to explore the moderating effects of sex, age, training status, and exercise performed. We also intended to explore a potential moderating effect for the muscle action type performed in testing (e.g., eccentric-only repetition, traditional eccentric-concentric repetition), but this was not possible given that only a small number of studies examined eccentric-only repetitions. For sex, we limited this to studies where groups were reported as male or female only (i.e., excluded mixed samples). We examined the mean age of the samples as a continuous predictor, but for ease of interpretation we present predicted values from this interaction model for 30, 50, and 70 years of age. For training status, we limited this to comparing those with and without prior resistance training experience as there were limited data for other populations (e.g., endurance trained) and for specific durations of prior resistance training experience. Last, we limited our examination of exercises to the bench press, chest press, squat, and leg press given that for these exercises we had more data available over a wider range of %1RM values, allowing comparison between upper- and lower-body exercise and between exercises involving similar muscle groups but different equipment (i.e., machines vs free weights). Results from the barbell squat were combined with results from the Smith machine squat, and results from the barbell bench press were combined with results from the Smith machine bench press. These data were combined because many papers did not include information on the equipment used, and of those papers that included such information, insufficient data were available to warrant exploration of separate REPS ~ %1RM relationships for Smith machine and barbell exercises. In each moderator interaction comparison, we calculated pairwise contrasts using ratios with 95% CIs given the use of log means and log SDs.

 Given the potential practical utility of the REPS ~ %1RM relationship, the statistical terminology used herein also warrants brief explanation to facilitate interpretation of the results. The number of repetitions performed at a given %1RM could be described by two parameters: a mean and an SD. The mean refers to the central tendency for repetitions performed by individuals, and the SD refers to the dispersion of repetitions performed. The point estimate for a given parameter refers to the best estimate of the parameter value in the population from which the sample was drawn, given the assumptions of the statistical model employed as an estimator and the sample data (in this case, the summary data from studies included in the meta-analysis described). Thus, when referring to the point estimate for either the mean repetitions or SDs in repetitions, we are referring to our best estimate of each of these parameters. However, we also present the uncertainty in our estimates for each of these, both mean and SD, by providing CIs from our estimator for each parameter. These are interpreted as being wide enough that a certain percentage of the time (95% in the present case), if we took samples (individual studies in this case) and employed a particular statistical model (meta-analysis in this case), we would expect them to include the true value of the parameter, given that the assumptions of the statistical model are met.

## Results

A total of 269 eligible studies were identified [1, 4, 6, 17, 36–84] [85–123] [124–216] [162, 217–267] [268–300]. These studies included 452 groups that contributed data from 952 repetitions-to-failure tests completed by 7289 individuals. The earliest study was published in 1961 and the latest in 2023. The descriptive characteristics of the groups in the identified studies are reported in the Electronic Supplementary Material (ESM) [see https://osf.io/r2xs7]. Results from 77 studies were extracted using WebPlotDigitizer.

The main descriptive results indicated that the samples (*k*) were predominantly male (*k* = 292; 66%), healthy (*k* = 433; 97%), < 59 years of age (*k* = 410; 92%; median of the mean age for samples 23 years), and resistance trained (*k* = 247; 60%). Barbells (*k* = 172; 47%), weight stack and plate-loaded machines (*k* = 145; 39%), and Smith machines (*k* = 33; 9%) were the most commonly used devices for testing. The most common exercises tested were the bench press (*k* = 189; 42%), leg press (*k* = 65; 14%), squat (*k* = 52; 12%), knee extension (*k* = 48; 11%), and chest press (*k* = 42; 9%). Testing was predominantly bilateral (*k* = 394; 89%) with repetition duration^1^ controlled using a metronome (*k* = 94; 68%) in those studies reporting it (though the majority did not report this; *k* = 311). Most studies involved tests using traditional concentric-eccentric repetitions (*k* = 439; 98%).

Not all identified repetitions-to-failure tests were included in the meta-analyses because effect sizes could not be calculated when variances were not reported. Further, we opted to only examine tests that had performed traditional concentric-eccentric repetitions as there was limited data for either concentric-only (1.1%) or eccentric-only tests (1.3%). It was possible therefore to include the results from 425 groups and 898 tests from 6970 individuals in our analyses. The median sample size for any included group was 13 participants with a range from 3 to 112 participants.

### Main Models

**Fig. 2:**
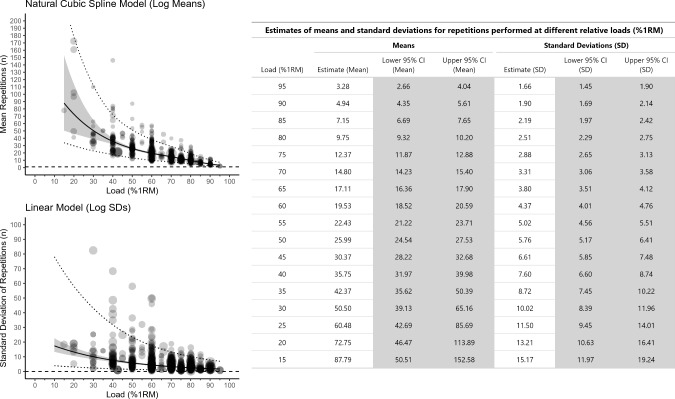
Meta-analytic scatterplots from main models for both the natural cubic spline model used to model log mean repetitions (top left panel) and the linear model used to model standard deviation (SD) of repetitions (bottom left panel). Estimates from both models have been exponentiated back to the raw repetitions scale. For the mean repetitions plot, the dashed horizontal reference line is at one repetition. For the SD of the repetitions plot, the dashed horizontal reference line is at zero. The grey band shows the 95% confidence interval (CI) and the dashed lines show the 95% prediction interval. A table showing the exact point estimates and 95% CIs for both mean repetitions, and SDs of repetitions, is presented that ranges from 15 to 95% 1 repetition maximum (RM) at 5% 1RM intervals (right panel)

Both of the main models exploring the relationships between %1RM and both mean repetitions and SD of repetitions indicated a negative trend in estimates with increasing %1RM. The mean number of repetitions decreases with increasing %1RM, as does the between-individuals SD in repetitions performed. Figure 2 presents the meta-analytic scatter plots for both the mean repetitions (natural cubic spline model of log means) and the SDs of repetitions (linear model of log SDs) with 95% CIs and 95% prediction intervals, alongside an updated REPS ~ %1RM table that ranged from 15 to 95% of 1RM in 5% intervals. The precision of estimates for both means and SDs are tight up to 65% 1RM range to ~ 1 repetition. Estimates from the models are less precise for lower %1RM values due to limited data at these loads.

### Moderators

**Fig. 3:**
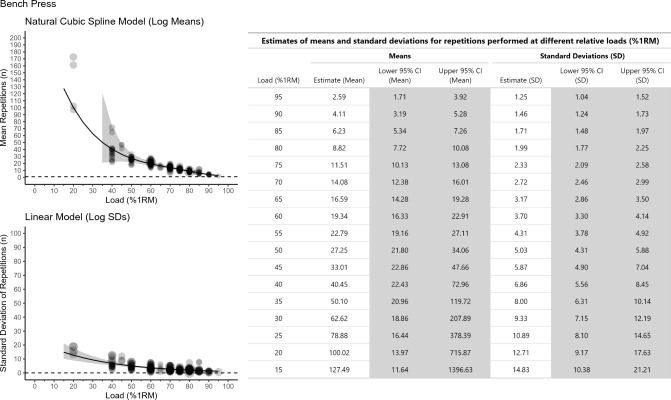
Meta-analytic scatterplots for the *bench press* for both the natural cubic spline model used to model log mean repetitions (top left panel) and the linear model used to model standard deviation (SD) of repetitions (bottom left panel). Estimates from both models have been exponentiated back to the raw repetitions scale. For the mean repetitions plot, the dashed horizontal reference line is at one repetition. For the SD of repetitions plot, the dashed horizontal reference line is at zero. The grey band shows the 95% confidence interval (CI). A table showing the exact point estimates and 95% CIs for both mean repetitions, and SD of repetitions, is presented that ranges from 15 to 95% 1 repetition maximum (RM) at 5% 1RM intervals (right panel)

**Fig. 4:**
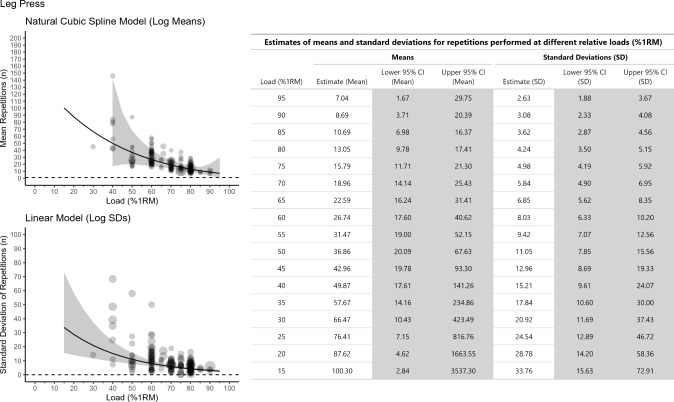
Meta-analytic scatterplots for the *leg press* for both the natural cubic spline model used to model log mean repetitions (top left panel) and the linear model used to model standard deviation (SD) of repetitions (bottom left panel). Estimates for both models have been exponentiated back to the raw repetitions scale. For the mean repetitions plot, the dashed horizontal reference line is at one repetition. For the SD of repetitions plot, the dashed horizontal reference line is at zero. The grey band shows the 95% confidence interval (CI). A table showing the exact point estimates and 95% CIs for both mean repetitions, and SD of repetitions, is presented that ranges from 15 to 95% 1 repetition maximum (RM) at 5% 1RM intervals (right panel)

The impact of most of the moderators was uncertain based on the precision of estimates for the contrasts. Whilst there were slight differences when comparing moderators such as sex (sex plot https://osf.io/xcesk, sex table https://osf.io/zmd8f), age (age plot https://osf.io/3tfxd, age table https://osf.io/mt7cs), training status (training status plot https://osf.io/kupbq, training status table https://osf.io/7964a), and exercise (exercise plot https://osf.io/kx6gp, exercise table https://osf.io/bxjh9) in point estimates for both mean and SD of repetitions, almost all interval estimates on contrast ratios included 1. Thus, it is uncertain if there are moderating effects for these variables in mean repetitions or SDs of repetitions (see contrast ratio tables for sex https://osf.io/jub3h, age https://osf.io/gavmc, training status https://osf.io/9f5ke, and exercise https://osf.io/kfbuh). The only exception was for contrasts between the bench press (Fig. 3) and leg press exercise (Fig. 4), where up to ~ 50% 1RM, fewer mean repetitions were possible in the bench press, and up to ~ 35% 1RM, there was also lower between-individuals SDs in number of repetitions possible for the bench press.

## Discussion

The purposes of this study were to use meta-regression to estimate the number of repetitions that individuals can be expected to complete at various percentages of the 1RM and to explore if the REPS ~ %1RM relationship is moderated by sex, age, training status, and exercise. From data collected on approximately 7000 individuals, we generated an updated main model table of the REPS ~ %1RM relationship (Fig. 2). Because sex, age, and training status did not clearly moderate the REPS ~ %1RM, the main model table can be used when prescribing resistance exercise to all individuals and for most exercises. However, differences in the REPS ~ %1RM relationship were observed for the leg press and bench press and thus separate tables were created for these two exercises. We were unable to explore muscle action type as a moderator owing to the lack of data available for repetitions-to-failure tests with eccentric-only muscle actions.

Our results update the REPS ~ %1RM table that has been presented in a commonly assigned strength and conditioning textbook for many years (Table 1) [7]. Table 1 provides only point estimates for the number of repetitions that an individual might be expected to complete at various percentages of the 1RM. Our updated table provides both mean repetition estimates, and estimates for between-individuals variation, and incorporates the uncertainty of these estimates by reporting their corresponding 95% CIs (Fig. 2).

As expected, we found that estimates for the mean number of repetitions decreased with increasing %1RM. Compared with Table 1, estimates in Fig. 2 are most different at lighter loads, whereas estimates at higher loads are more similar between Table 1 and Fig. 2. For example, in Table 1, estimates at 90% and 70% 1RM are 4 and 11 repetitions, respectively. In Fig. 2, estimates at 90% and 70% 1RM are ~ 5 and ~ 15 repetitions, respectively. For the bench press, estimates in Fig. 2 are generally similar with Table 1. For example, at 90, 80, and 70% 1RM, the estimates in Table 1 are 4, 8, and 11 repetitions, respectively. In Fig. 2, the estimates at these same relative loads are ~ 4, ~ 9, and ~ 14 repetitions, respectively. However, estimates for the leg press are notably higher in Fig. 2 than Table 1. At 90, 80, and 70% 1RM, point estimates in Fig. 2 are ~ 9, ~ 13, and ~ 19 repetitions, respectively.

In addition to the estimates for mean repetitions, Fig. 2 provides estimates for SDs for repetitions between individuals. This advances Table 1, which does not account for between-individual variability in test performance. The estimates for SDs also increases as %1RM decreases. For example, at 80% 1RM, the estimate for the SD about the point estimate is 2.51 repetitions, whereas at 60% 1RM the estimate is 4.36 repetitions. These results reveal greater between-individual heterogeneity in repetitions completed at lighter than heavier relative loads. Why between-individual heterogeneity in repetitions completed is greater at lighter loads is not entirely clear. This result may reflect the commonly observed mean–variance relationship (i.e., as means increase so do their corresponding SDs) that has been reported for other exercise outcomes such as muscle strength [301]. Our exploratory meta-regression model confirmed the presence of such a mean–variance relationship (see https://osf.io/sknyr). This variance could also be influenced by heteroskedasticity in measurement error whereby it also scales with measured repetitions. Thus, although large SDs could be due to between-individual heterogeneity in repetitions completed, a mathematical phenomenon, or heteroskedastic measurement errors, this information is still practically useful because it illustrates the amount of variance that can be expected.

We thought that sex and age might moderate the REPS ~ %1RM relationship because of evidence suggesting that sex [8–11] and age [12–14] impact muscle fatigability. However, the REPS ~ %1RM relationship was largely similar between men and women, and the relationship was also similar between younger and older adults, potentially questioning the magnitude of the impact of these factors on fatigability. Consequently, we did not generate sex- or age-specific REPS ~ %1RM tables.

We also examined exercise as a potential moderator of the REPS ~ %1RM relationship. We observed a difference in the REPS ~ %1RM relationship between the leg press and bench press, with greater mean repetitions completed in the leg press than bench press across the spectrum of relative loads. For example, at 80% and 70% 1RM, the estimated number of repetitions in the leg press were 13.1 [95% CI 9.8–17.5] and 19.0 [95% CI 14.2–25.5], respectively, whereas for the bench press, the estimated number of repetitions were 8.8 [95% CI 7.7–10.1] and 14.1 [95% CI 12.4–16.1], respectively. Consequently, we generated separate REPS ~ %1RM tables for the bench press (Fig. 3) and leg press (Fig. 4). For all other exercises, the main model table is most applicable (Fig. 2).

We also intended to explore if the REPS-%1RM relationship differs between concentric and eccentric muscle actions. However, only 1% of all data were from eccentric-only testing. Consequently, we could not determine whether different REPS ~ %1RM tables should exist for eccentric-only and traditional repetitions. Results from a small number of studies suggest that at equal *relative* loads, more eccentric-only than concentric-only repetitions can be completed at certain relative loads for some exercises [17, 169, 302]. If these results are replicated in future research, a REPS ~ %1RM table specific to eccentric muscle actions will be needed, particularly as eccentric resistance exercise is growing in popularity and new technologies are making its prescription more feasible [11, 18, 19].

### Limitations and Future Research

The current study is not without limitations. Our search strategy did not follow standard guidelines for meta-analyses. The disadvantage of this strategy is that it makes future attempts to replicate the strategy challenging or impossible. Nevertheless, our search identified 269 studies, which is substantially more studies than the current REPS ~ %1RM table is based on (Table 1) [7]. Moreover, all references, analyses, and results from the current study have been made publicly available. Researchers are welcome to use the publicly available information to further explore the data or build from it. A second limitation of the current study is that the amount of data available did not permit formal analyses that might be of interest to some exercise practitioners, for example, whether the REPS ~ %1RM relationship differs between different types of athletes [92, 202, 237]. Some results suggest endurance athletes can perform more repetitions at loads ≤ 75% 1RM than can strength-power athletes [92, 202, 237]. Moving forward, the solution to such limitations is to collectmore data. More data is needed to provide more precise point estimates of the number of repetitions that individuals can be expected to complete across the relative loading spectrum. Most data from the REPS ~ %1RM relationship have been collected on healthy individuals who are aged 20–40 years. Thus, future research can examine the REPS ~ %1RM relationship in older adults, patient groups, and specific athlete groups. Future research can also explore the REPS ~ %1RM relationship for exercises that are commonly prescribed but for which minimal data are available (e.g., overhead press, lateral pulldown, seated row, triceps extension, knee flexion, calf raise). Last, only a relatively narrow range of repetition durations were reported with the magnitude of their impact being relatively small and uncertain. As some resistance training protocols employ long repetition durations and low repetition numbers (e.g., 6 repetitions at 10-s concentric and 10-s eccentric) [303], the REPS ~ %1RM relationship might differ at more extreme repetition durations, and thus further research can explore this topic.

## Conclusions

The REPS ~ %1RM relationship is foundational knowledge in resistance exercise programming. It gives practitioners a sense of the relative loads that can be prescribed to allow for a certain number of repetitions to be completed. Though a general table of the REPS ~ %1RM relationship has been available for many years (Table 1), it has not incorporated uncertainty into point estimates or accounted for between-individuals variation in performance. We updated this table. After using meta-regression to analyze all available literature on repetitions-to-failure tests, we generated a main model table of estimates for mean repetitions *and* SDs and 95% CIs around the point estimates across the relative loading spectrum (Fig. 2). This table can be used to guide resistance exercise prescriptions for all individuals and for most exercises. However, because significantly more repetitions can be completed in the leg press than the bench press, separate tables should be referenced when prescribing resistance exercise for these two exercises (Figs. 3 and 4). Future research involving hundreds, if not thousands, of participants will be necessary to establish precise REPS ~ %1RM relationships for other exercises and specific populations.

## Footnotes

1 As repetition duration might impact the REPS ~ %1RM relationship, we included an exploratory analysis of this in studies where the repetition duration was reported. The range for reported total repetitions durations (i.e., both concentric and eccentric phases) was 1.4–6.0 s coming from only 122 of the included studies (46%). Whilst there was a tendency for fewer repetitions to be performed when using longer repetition durations, almost all interval estimates on contrast ratios included 1 and thus it is uncertain what the exact extent of moderating effects for this variable is over this range upon mean repetitions or SDs of repetitions (see ESM for figure https://osf.io/e9y7h, estimates table https://osf.io/yjrwz, and contrasts table https://osf.io/yje3k).

## Declarations

### Availability of Data and Material

The extracted data, and the code used to analyze that data, are available as supplementary materials at the Open Science Framework (https://osf.io/s94gf/).
